# The Financial Problem of the Voluntary Hospitals

**Published:** 1909-05-22

**Authors:** 


### HOSPITAL ADMINISTRATION.

### CONSTRUCTION AND ECONOMICS.

### THE FINANCIAL PROBLEM OF THE VOLUNTARY HOSPITALS.

POSSIBLE EFFECTS OF THE BUDGET.

Those who are responsible for raising the money for hospital purposes in this country must regard the Budget introduced by Mr. Lloyd George with something like dismay. With the exception of two types of hospital financiers, those who understand their business properly have been encouraged rather than depressed of late years by the results of their efforts. It is not a little remarkable that, excluding legacies, the ordinary income of 796 voluntary hospital in 1906 should shew an increase of upwards of £100,000 compared with the previous year, the total sum raised in ordinary income in 1906 amounting to no less than £2,558,000 in round figures. This ordinary income has increased from £1,931,000 in 1896 to £2,558,000 in 1906, an increase of 33 per cent. The total income from all sources has increased in the ten years from £2,528,000 to £3,673,000, an increase of 45 per cent., although the yield from legacies in the latter year only shews an increase of 13 per cent. compared with the receipts from this source in 1896. These figures afford striking testimony to the liberality of the public where hospitals are concerned. This popularity has greatly and deservedly increased of late years, owing to a continuous increase in the average efficiency throughout the country. The increase in the yield of contributions from voluntary sources is, however, largely due to the extension of the area of givers, owing mainly to the work of the League of Mercy, which has now some 15,000 honorary workers constantly engaged in pressing the claims of the hospitals mainly upon the smaller givers, and of the steady growth in public confidence in the administration of metropolitan hospitals, owing to the establishment of the King's Fund.

It may be justly said, in view of the figures and results just recorded, that hospital managers should have relatively little anxiety in regard to their revenues. The last twelve years have been years of depression, especially in the metropolis, and it is very remarkable that, despite this fact, the yield in voluntary income should have steadily and largely increased. But for this very reason, especially when we take the sources of revenue and examine them in detail, those responsible for the management of the voluntary hospitals must regard the present Budget with feelings of grave alarm. The Hospital has nothing to do with politics, and we propose to deal with the Budget from a business point of view entirely, as the great City merchants who signed the protest against it have dealt with it. The mischief of the Budget is that it has written all over it startling evidence that many matters have been introduced, the bearing of which has not been grasped, if it has even been seriously studied by the present Chancellor of the Exchequer. He has succeeded, largely owing to this fact, in excit[illegible] universal misgiving amongst the very people [illegible] form the backbone for revenue purposes not onl[illegible] the hospitals but of the nation. Serious men [illegible] not interfere with their own business in this hurr[illegible] careless manner, and they feel that such treatm[illegible] of national finance is a dangerous novelty in [illegible] history of British Budgets, the meaning and nat[illegible] of which prudent men cannot estimate. So [illegible] gravest uneasiness has been caused where such feeling might and ought to have been prevent[illegible] by the exercise of adequate care on the part of [illegible] Chancellor of the Exchequer, who would so h[illegible] worthily followed in the footsteps of the many gre[illegible] men who have preceded him in this important offic[illegible] No more striking proof of hurry and inadequa[illegible] attention and knowledge on the part of the prese[illegible] Chancellor of the Exchequer could be afford[illegible] than the fact that it is currently believed, and [illegible] evidence on this point is striking, that Mr. Ll[illegible] George himself does not yet know the nature [illegible] bearing of the clauses in the Financial Act bas[illegible] upon the Budget, which has yet to be introduc[illegible] Hence prudent people are alarmed, as we have sa[illegible] and when a prudent business man is alarmed ab[illegible] money, he cuts down his expenditure and curta[illegible] it in every possible direction. It is well known [illegible] in regard to bills many people have acquire[illegible] habit of putting the doctor's account at the bo[illegible] of the file on the ground that he is a memb[illegible] a great profession and cannot possibly be in [illegible] of money. So too the majority of people, [illegible] they are pinched for funds, economise by cu[illegible] off charitable gifts or reducing them to a mini[illegible] Should the present alarm created by the B[illegible] continue there is no question that the revenu[illegible] the hospitals from voluntary sources must be great[illegible] reduced in the course of the present year.

The vulnerability of hospital revenues will [illegible] come apparent when we state that 114 of the gr[illegible] general hospitals in the United Kingdom deri[illegible] 21 per cent. of their ordinary income from ann[illegible] subscriptions, the yield in London from this sou[illegible] being only 12 per cent. Fifteen per cent. came fr[illegible] donations and boxes against 19 per cent. [illegible] London, Hospital Sunday produced 6½ per ce[illegible] Hospital Saturday 4 per cent., and workpeopl[illegible] contributions 8¼ per cent. It is worthy of note t[illegible] in London the yield from Hospital Saturday is o[illegible] 1½ per cent. and from workpeople's contributi[illegible] only ¼ per cent., that is to say one thirty-seco[illegible] of the actual sum received from this source by p[illegible] vincial, Scotch and Irish hospitals. The case [illegible] special hospitals throughout the United Kingd[illegible] is equally serious, the yield from various volunta[illegible] sources during 1907 being as follows: annual su[illegible] scriptions 26 per cent., donations and boxes 22 [illegible] cent., Hospital Sunday 7 per cent., Hospital Satu[illegible]

y 2 per cent., and workpeople's contributions $1\frac{3}{5}$ [illegible] cent. In London the general hospitals received [illegible] per cent. of the King's Fund, whilst the special [illegible]spitals received $11\frac{1}{4}$ per cent. from the same [illegible]urce. These figures should be noted, because it [illegible]as been stated in the *Medical Press* and elsewhere [illegible]at the awards of the King's Fund have shewn a [illegible]rked unfairness to special hospitals. These [illegible]ures emphatically prove the falsity and un[illegible]sonableness of any such statement. It is well to [illegible]e too that Hospital Saturday only produced $1\frac{1}{2}$ [illegible] cent. of the income of the great general hospitals [illegible] London, compared with 4 per cent., the average [illegible]ld from this source as represented by the [illegible]ounts of 114 of the great general hospitals in the [illegible]ited Kingdom.

[illegible]e may add that the 114 great general hospitals [illegible] the United Kingdom derived 35 per cent. [illegible] their income from invested property, whereas [illegible] per cent. of the revenue of London hospitals [illegible]mes from this source. The invested funds of [illegible]ecial hospitals are much smaller, the annual [illegible]eld from this source having been $18\frac{1}{2}$ per [illegible]nt. from the whole country and 15 per cent. from [illegible]e metropolis. Sixty per cent. of the revenue of the hospitals of the United Kingdom is derived from voluntary sources, and the greater part of this might easily become forfeit should the public alarm created by the present Budget prove well founded.

Whether it is or not the crystallised proposals of Mr. Lloyd George, as embodied in the Finance Bill, which is awaited with grave anxiety by men of business in all parts of the country and of all shades of political opinion, will testify. We cannot in any case forget that his proposals so far as they are at present understood have created grave apprehension throughout the business world in the minds of the very classes who in the past have supplied the resources upon which the voluntary system of hospital support depends for its maintenance. If these resources were withdrawn the taxpayers would have to find, as the figures we give at the commencement of this article prove, at least two millions a year for the maintenance of hospitals, and in a few years, should they become State institutions, there is little doubt that the taxpayers would have to find from four to five millions annually, or the sick and suffering poor of these islands must be deprived of much of the medical aid and comforts which they now enjoy under the voluntary system.

